# Effective Pro-Inflammatory Induced Activity of GALT, a Conserved Antigen in *A. Pleuropneumoniae*, Improves the Cytokines Secretion of Macrophage via p38, ERK1/2 and JNK MAPKs Signal Pathway

**DOI:** 10.3389/fcimb.2018.00337

**Published:** 2018-09-25

**Authors:** Fei Zhang, Qin Zhao, Jin Tian, Yung-Fu Chang, Xintian Wen, Xiaobo Huang, Rui Wu, Yiping Wen, Qigui Yan, Yong Huang, Xiaoping Ma, Xinfeng Han, Chang Miao, Sanjie Cao

**Affiliations:** ^1^College of Veterinary Medicine, Research Center of Swine Disease, Sichuan Agricultural University, Chengdu, China; ^2^Department of Population Medicine and Diagnostic Sciences, College of Veterinary Medicine, Cornell University, Ithaca, NY, United States; ^3^National Teaching and Experimental Center of Animal, Sichuan Agricultural University, Chengdu, China; ^4^Sichuan Science-Observation Experimental Station of Veterinary Drugs and Veterinary Diagnostic Technology, Ministry of Agriculture, Chengdu, China

**Keywords:** *Actinobacillus pleuropneumoniae*, pro-inflammatory cytokines, MAPKs pathway, innate immune system, toll-like receptors

## Abstract

GALT is a highly conserved antigen in gram-negative bacteria, and has been shown to play a crucial role in the pathogenesis of many zoonoses. *Actinobacillus pleuropneumoniae* (APP) is a widespread respiratory system pathogen belonging to the *Pasteuriaceae* family. The functional mechanisms of GALT in the process of infection remain unclear. The aim of this study is to analyze roles of GALT in the pathogenesis of APP infection. Recombinant GALT was expressed in *E. coli*, purified, and was used to treat a Raw 264.7 macrophage line. Stimulation of Raw 264.7 macrophages with recombinant GALT protein induced the expression of pro-inflammatory cytokines (TNF-α, IL-1β, and IL-6). Compared with negative control, GALT led to increased production of pro-inflammatory cytokines in treated cells. Furthermore, specific inhibitors of the extracellular signal-regulated P38 and JNK MAPKs pathways significantly decreased GALT-induced pro-inflammatory cytokine production, and a western blot assay showed that GALT stimulation induced the activation of the MAPKs pathway. This process included cell-signaling pathways like P38, ERK1/2 and JNK MAPKs, and NF-κB. Both TLR2 and TLR4 were receptors of GALT antigens, whereas they played negative and positive roles (respectively) in the process of induction and expression of pro-inflammatory cytokines. Taken together, our data indicate that GALT is a novel pro-inflammatory mediator and induces TLR2 and TLR4-dependent pro-inflammatory activity in Raw 264.7 macrophages through P38, ERK1/2, and JNK MAPKs pathways.

## Introduction

*Actinobacillus pleuropneumoniae* (APP) is an important bacterial respiratory tract pathogen, which causes infectious pleural pneumonia in pigs and is highly communicable. APP is a gram-negative bacterium that infects piglets; infection is characterized by cellulitis and hemorrhagic necrotizing pneumonia and is readily contagious. Pathogenic bacteria invade the host, typically via colonization of specific tissues. Upon proliferation, invading bacteria often secrete significant amounts of various exotoxins; the majority of these are readily soluble and disperse to act on various target host cells lines or tissues. During the infection process, large numbers of bacteria die; the disintegration of bacterial cells may release various virulence factors and/or bacterial components that trigger host immune responses. APP infection elevates the secretion of pro-inflammatory cytokines which are crucial factors in mediating and regulating inflammatory responses to APP infection (Choi et al., 1999). At the present time, studies have focused on bacterial virulence factors interacting with the host tissues.

Recognition of microbes by the host innate immune system is a crucial step in defense against microbial infection (Iwasaki and Medzhitov, 2010; Sasai and Yamamoto, 2013). Immune responses are often mediated by interactions between pathogen cell surface antigens and immune cell receptors, such as toll-like receptors (TLRs). TLRs were discovered in the mid-1990s (Medzhitov et al., 1997). Bacteria, viruses, fungi and parasites have been shown to be recognized by TLR2 (Aliprantis et al., 1999; Kurt-Jones et al., 2000; Sasai and Yamamoto, 2013). TLRs were found to induce activation of the NF-kB pathway and expression of genes for the inflammatory cytokines IL-1 and IL-6 (Medzhitov et al., 1997). TLR4 is essential in the recognition of and response to antigenic components of gram-negative bacteria (Sasai and Yamamoto, 2013).

Pattern recognition receptors (PRRs) of innate immune system are well characterized as microbial sensors to detect invariant molecular patterns (Iwasaki and Medzhitov, 2010). Pathogen-associated molecular patterns (PAMPs) are conserved molecular patterns which are shared with a large group of microorganisms (Akira et al., 2001). Toll-like receptor (TLR) family consists of transmembrane PRRs, which express on the plasma membrane (Takeda and Akira, 2005). As PRRs, TLRs are of huge importance in the recognition of microbial components (Akira et al., 2001). Mitogen-activated protein kinase (MAPK) and nuclear factor-κB (NF-κB) pathways are significant contributors in immune responses, both of which are activated by PRRs (Kawai and Akira, 2010).

Identification and characterization of virulence factors in a given pathogen (like APP) are crucial in developing strategies for combating or controlling diseases that said pathogen causes. Many research tools are now available for efforts in studying pathogen virulence factors (Fuller et al., 2000; Handfield et al., 2000; Vigil et al., 2011). *In vivo*-induced antigens play a critical role in infections by pathogenic microbes. These antigens facilitate colonization of pathogens and adaptation to the host. GALT is an *in vivo*-induced antigen of APP identified in previous study using IVIAT (Zhang et al., 2015a). The *gal*T gene is located in the *gal* gene cluster, which is involved in the metabolism of galactose. Genes of this cluster are widely distributed in various pathogenic agents and these genes have been demonstrated to be associated with the virulence factors such as formation of LPS, CPS, and biofilm formation (Priebe et al., 2004; Wong and Akerley, 2005; Ramjeet et al., 2008; Chai et al., 2012; Caboni et al., 2015; Meyer et al., 2015; Oechslin et al., 2017; Pires et al., 2017). However, the mechanism of GALT-mediated pathogenesis is still unknown.

Our previous studies on *in vivo*-induced antigens of APP demonstrated that the expression of these antigens were up-regulated significantly during infection; some of these *in vivo*-induced antigens have shown to be highly immunogenic, and have high potential as vaccine candidates, which induce both innate and adaptive immune responses (Zhang et al., 2015a, 2016). Therefore, an in-depth study of APP pathogenesis and immune mechanisms are warranted for the development of safe and effective vaccines and therapeutic drugs. Previous studies indicated that the protein is highly immunogenic and can stimulate immune protection against pathogenic microbes. The aim of this study is to dissect the mechanism of inflammatory response to GALT protein exposure. Nevertheless, the function of GALT in the progress of infection is not well understood. In this study, macrophages were treated with GALT and RT-PCR or ELISA were used to analyze for various cytokine expression. As an significant mediator of the inflammatory response, macrophages are one of the crucial cell types to study the signaling pathways that regulate immunity (Medzhitov and Horng, 2009). In the present study, MAPKs and NF-κB signaling-pathway have been studied, including extracellular signal-regulated kinase 1/2(ERK1/2), P38, P65, and Jun N-terminal kinase 1/2 (JNK1/2) in the context of innate immunity.

## Materials and methods

### Bacterial strains and cultural condition

APP strains were cultured in Trypticase Soy Agar (TSA) or Trypicase Soy Broth (TSB) with 5% (v/v) fetal bovine serum (FBS) and nicotinamide adenine dinucleotide (NAD; 15 mg/mL) was added. The protein expression clones of *E.coli gal*T (BL21) were constructed and stored in research center of swine disease of Sichuan agricultural University (Zhang et al., 2016). GALT protein expression strain was cultured in LB broth and agar, 37°C. Kanamycin was added to 50 μg/ml concentration.

### Expression and purification of recombinant GALT protein

In previous studies, we found that GALT is an *in vivo*-induced antigen(Zhang et al., 2015a, 2016). The pET-*gal*T (BL21) clone was recovered in LB medium with 50 μg/ml Kanamycin grown in a small overnight (o/n) culture. This was added as a seed for a larger expression culture (100x o/n culture voL). When the OD_600_ of the expression culture reached 0.6, 1 mM IPTG (isopropyl-b-D- thiogalactopyranoside) was added into the medium and the culture was induced for another 4 h. The culture was centrifuged and the cells were collected and washed using cold PBS. Collected cells were sonicated for lysis. The his-tag fusion recombinant protein was purified by Ni-NTA agarose chromatography. Purified recombinant protein GALT was analyzed by SDS-PAGE electrophoresis.

To exclude endotoxin contamination in the purified protein, EtEraser Endotoxin Removal Kit (Xiamen Bioendo Technology Co.,Ltd., China) was used to treat the purified protein (Peddireddy et al., 2016). Chromogenic End-point LAL assay (Xiamen Bioendo Technology Co.,Ltd., China) was used to quantify the endotoxin. Concentration of recombinant protein was detected using the BCA method. The GALT protein treated as above was stored at −80°C until use.

### Indirect immunofluorescence

Indirect immunofluorescence was performed as previously described with necessary modify (Yang et al., 2014). APP strain MS71 and L20 were cultured in TSB. After washed and resuspended in PBS, the bacteria were fixed with 1% paraformaldehyde for 20 min at 25°C. After centrifuged 5 min at 8,000 g, bacteria were washed with PBS and resuspended in PBS containing 10% FBS. Serum was added to the tubes with a incubation at 37°C for 1 h. GALT immunized mice serum was used to detect the GALT antigen on the cell surface of APP strain MS71 and L20. Negative mice serum and inactivated APP L20 immunized mice serum was added as negative and positive control respectively. Bacteria were washed again with PBS and FITC-labeled Goat Anti-Mouse IgG (H+L) (Earthox, USA) antibodies was added to detect the bound antibodies. Then, bacteria were washed with PBS and resuspended in PBS containing 3% bovine serum albumin (BSA) in darkness. The samples were observed by fluorescence microscopy ECLIPSE 80i (Nikon, Japan).

### Cell culture

Mouse monocyte-macrophage leukemia cell line Raw 264.7 was maintained in our laboratory. Macrophage Raw 267.4 was cultured in complete RPMI1640 (10% fetal calf serum added). Cells were cultured in 5% CO2 at a temperature of 37°C. Macrophages were added to 12-well cell culture plates (1 mL/well) at 1 × 10^6^ cells per well. To minimize the contamination of lipopolysaccharide (LPS), polymyxin B (PMB) was added at a concentration of 10 μg/mL throughout the experiments as previously described (Chen et al., 2011; Wu et al., 2011).

### Detected of pro-inflammatory cytokines induced by GALT

GALT was added to Raw 264.7 macrophage culture medium at a final concentration of 10 μg/mL; the positive control culture had 200 ng/ml LPS (LPS medium without polymyxin B). The negative control had no protein added. The cultures were incubated for 12 h, then spun down. The cell culture supernatant was collected in aliquots stored at −70°C until ELISA screening. Cell culture supernatants were collected and the concentrations of cytokines IL-1β, IL-6, and TNF-α were detected using an ELISA kit (eBioscience, USA) according to the instruction manual.

### Real-time RT-PCR

Raw 264.7 cells were treated with GALT protein (10 μg ml-1); untreated cells served as a negative control, and LPS at a final concentration of 200 ng/mL was the positive control. All treated cultures were set to incubate for 12 h. Total RNA was extracted using RNA extraction solution (Servicebio, China). Tips and tubes were sterilization to eliminate RNase contamination. Culture supernatant fluid was removed thoroughly and cells were washed with 1 ml 4°C PBS. RNA extracting solution was added; samples shaken gently to lyse cells. Pellets were lysed with RNase-Free water and treated with DNase (Promega, Madison, WI, USA) to clear potential DNA contamination. Lysed solution was transferred into 1.5 ml centrifuge tubes. Two hundred and Fifty micro liter trichloromethane was added to the samples and mixed thoroughly. After 3 min standing, the tubes were centrifuged for 10 min at 4°C, 12,000 rpm. Supernatants were transferred to another tube. A 0.8 volume isopropanol was added and samples inverted several times to mix. After 15 min standing at −20°C, samples were centrifuged 10 min in 12,000 rpm at 4°C. After centrifugation, supernatant was removed and the white pellet in the bottom of the tubes was washed using 75% ethanol. Samples were centrifuged for 10 min at 12,000 rpm at 4°C and pellets were dried on the benchtop. RNA was dissolved in 15 μl RNase-Free water. After 55°C incubation, purity and concentration were measured using a spectrophotometer Nanodrop 2000 (Thermo, USA) at 260 nm. Concentration of the total RNA was adjusted to 200 ng/μl. RT-PCR was performed using RevertAid First Strand cDNA Synthesis Kit (Thermo, USA). RT-PCR was conducted using RevertAid First Strand cDNA Synthesis Kit (Thermo, USA). Two microgram RNA and one micro liter oligo(dT)18 were added into one PCR tube. HyPure ^TM^Molecular Biology Grade Water (HyClone, USA) was added to bring the volume of the solution up to 12 μL. Tubes were incubated at 65°C for 5 min on PCR thermocycle instrument and then placed on ice immediately. Four microliter 5 × Reaction Buffer, 2 μL 10 mM dNTP Mix, 1 μL RiboLock RNase inhibitor (20 U/μL) and 1 μL RevertAi M-MuLV RT were added subsequently and then mixed thoroughly. Thermocycler parameters were set at 42°C for 60 min and 70°C for 5 min to inactivate Reverse transcriptase. The reaction ingredients for qPCR were 12.5 μl of 2× qPCR Mix (FastStart Universal SYBR Green Master, Roche, Switzerland), 2.0 μl of 7.5 μM primers, 2.5 μl of RT products, 8.0 μl of dd H_2_O. The primers for q RT-PCR used in this study are shown in Table 1. Amplification parameters were as follows: Denaturing at 95°C for 10 min, 40 cycles at 95°C, 15 s; annealing 60°C for 60 s and extension at 70°C for 1 min. Relative quantification analysis was performed using ΔΔCT method(Kats et al., 2016) to analyze the level of cytokines expression (Table 1). Real-time PCR was used to analyze the relative expression of IL-1β, IL-6 and TNF-α. β-actin was chosen to be a reference gene in a relative quantification analysis (Tang et al., 2014).

**Table 1:** The sequences of primers used in qRT-PCR analysis.

**Gene**	**Primer sequences**
IL-1β	F 5′-CAGGATGAGGACATGACACC-3′
	R 5′-CTCTGCAGACTCAAACTCCAC-3′
IL-6	F 5′-GTACTCCAGAAGACCAGAGG-3′
	R 5′-TGCTGGTGACAACCACGGCC-3′
TNF-α	F 5′-TTGACCTCAGCGCTGAGTTG-3′
	R 5′-CCTGTAGCCCACGTCGTAGC-3′
β-actin	F 5′-GTGGGCCGCCCTAGGACCAG-3′
	R 5′-GGAGGAAGAGGATGCGGCAGT-3′

### TLR2 and TLR4 blocking assay

Macrophage recognition receptor of GALT was studied. LEAF™ Purified anti-mouse TLR4 (CD284)/MD2 Complex (BioLegend) and Ultra-LEAF™ Purified anti-mouse/human CD282 (TLR2) Recombinant (BioLegend) were used to block the corresponding Toll-like receptor. Both of these two antibodies were added to a final concentration of 8 μg/mL. GALT protein was added to cultured macrophages to a final concentration of 10 μg/mL for 12 h after 30 min pre-treatment with two blocking antibodies. Cell culture supernatant was collected after GALT treatment for detection of cytokines (IL-1β, IL-6, IFN-α) using indirect ELISA (Invitrogen, USA). Results were analyzed to determine cytokine response levels and to confirm identity of receptors contributing to the response to GALT exposure.

### Western blot analysis

Raw 264.7 cells were stimulated with GALT (10 μg/mL) for 12 h. These were then washed with cold PBS and were collected by centrifugation. The total protein of GALT-treated and untreated macrophages was extracted. The collected cells were resuspended in phosphate buffered saline (Roche) and RIPA lysis buffer and allowed to sit in an ice bath for 15 min. Western blot was conducted to analyze GALT induced signal transduction residue phosphorylation. After stimulation with GALT (10 μg/mL) for 10 h, protein was extracted from treated cells. Appropriate volumes of RIPA (plus protease inhibitor) were added to the cultures; these were incubated on a plate shaker for 5 min. Cells were moved from the culture plates and collected in 1.5 ml centrifuge tubes. Tubes were incubated on ice; cells were pipetted several times to ensure thorough lysis. Lysate was centrifuged at 12,000 × g for 5 min and supernatant, which contained the desired protein, was collected. Concentration of the protein solution was quantified by BCA method. Fifty microgram lysed protein were analyzed by 12% SDS-PAGE and the protein was transferred onto 0.22 um PVDF membrane by wet transfer. The membranes were blocked with 5% skim milk at room temperature for 1 h. Blocked membranes were reacted with specific antibodies at 4°C overnight. Membranes were washed 3 times using TBST, 5 min per wash. Subsequently, these proteins were probed with specific Abs against P38, P-P38, Erk l/2, p-Erk l/2, JNK, P-JNK, P65, P-P65, TLR2, and TLR4 (Cell Signaling Technology). The anti-TLR-2 (or TLR-4) antibodies were then expressed as p-Erk l/2, Erk l/2, p-p38, P38, pJNK, JNK, (Santa Cruz Biotechnology Inc., USA) for 2 h followed by three washes with PBS. Secondary antibody labeled with horseradish peroxidase was added and incubated for 2 h at room temperature, then washed three times with PBS. Chemiluminescence reagent (ECL) was added to the PVDF membrane, and images of the reaction were recorded. Band luminosity intensity was quantified using Image J software using β-actin as an internal control. After 3 washes with TBST, membrane was reacted with ECL. Alpha software was used for analysis of Integrated Optical Density.

### Effect of inhibitor on the MAPKs signal pathway

Raw 264.7 macrophages (1 × 10^6^ cells ml^−1^) were cultured in 12-well cell culture plates. Cells were pretreated with the following specific inhibitors for 30 min prior to GALT addition: SB203580 (p38 inhibitor; 10 μM), U0126 (for ERK1/2; 10 μM), SP600125 (JNK inhibitor; 10 μM), PDTC (PDTC; for NF-κB; 20 μM) (Table 2). Inhibitors for studying signal pathways were purchased from Beyotime (China). Cell culture supernatant was sampled at the indicated times; these were stored at −80°C. Supernatants collected were detected for the concentration of IL-1β, IL-6, and TNF-α using ELISA kit.

**Table 2:** The inhibitor experiment.

**Group**	**GALT(12 μg/mL)**	**DMSO(1 μl/mL)**	**(SB203580) (p38 inhibitor) (10 μM)**	**(U0126) (an ERK1/2 inhibitor) (10 μM)**	**(SP600125)(JNK inhibitor)**	**(PDTC) (NF-kB inhibitor) (10 μM)**	**Polymyxin B**
1	–	–	–	–	–	–	+
2	+	–	–	–	–	–	+
3	+	+	–	–	–	–	+
4	+	–	+	–	–	–	+
5	+	–	–	+	–	–	+
6	+	–	–	–	+	–	+
7	+	–	–	–	–	+	+

### Bioinformatics analysis

Amino acid sequences of GALT of APP and other pathogens were retrieved from Genbank. Amino acid sequences of GALT from 14 different bacteria were subjected to PredictProtein (Yachdav et al., 2014) (https://open.predictprotein.org/) for sequence analysis. The program BLAST-P was used to detect similar amino acid sequences (https://blast.ncbi.nlm.nih.gov/Blast.cgi) of GALT. MegAlign was used to analyze sequence distances among different microbes (By Jotun Hein method). Online server PredictProtein (https://open.predictprotein.org/) was used to analyze the amino acid sequences.

### Statistical analysis

All data in present study are expressed as mean ± standard deviation. Statistical significance analysis was performed using Student's *t* test of GraphPad Prism software (San Diego, CA, USA). The bars represent the standard errors of the means, based on three independent experiments. In the results of data comparison analysis in present study, p < 0.05 was considered as significant difference. “^**^” indicates *P* < 0.01, “^***^” indicates *P* < 0.001 and “ns” indicates not significant.

## Results

### Purified GALT protein could be applied on cell treatment

**Figure 1:**
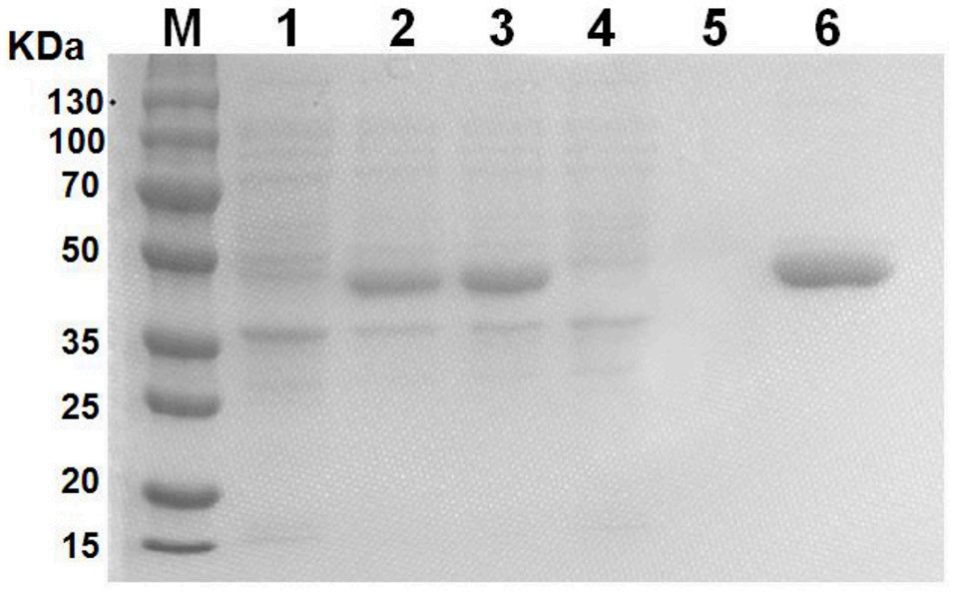
Expression and purification of recombinant GALT protein. M, protein marker, 1, BL21(pET-28a), 2, Uninduced GALT(BL21), 3, GALT(BL21) induced by IPTG for 4 h, 4, BL21(pET-28a) induced by IPTG for 4 h, 5, Results of purification of induced BL21(pET-28a), 6, Purified recombinant GALT protein.

GALT protein was expressed and purified *in vitro* as an antigen to investigate the effect on macrophages. The products of induction and purification were analyzed using SDS-PAGE (Figure 1). *E.coli* BL21 strains harbored the pET-28a vector alone to serve as control. The GALT protein was successfully expressed in BL21, which was induced using IPTG for 4 h. Affinity chromatography was used to purify the recombinant GALT and a single high purity band was detected by SDS-PAGE. After endotoxin removal, the concentration of endotoxin was 0.5 EU (endotoxin unit)/mL in the purified GALT, which was subsequently used to treat macrophages.

### GALT could be detected on the cell surface of APP

**Figure 2:**
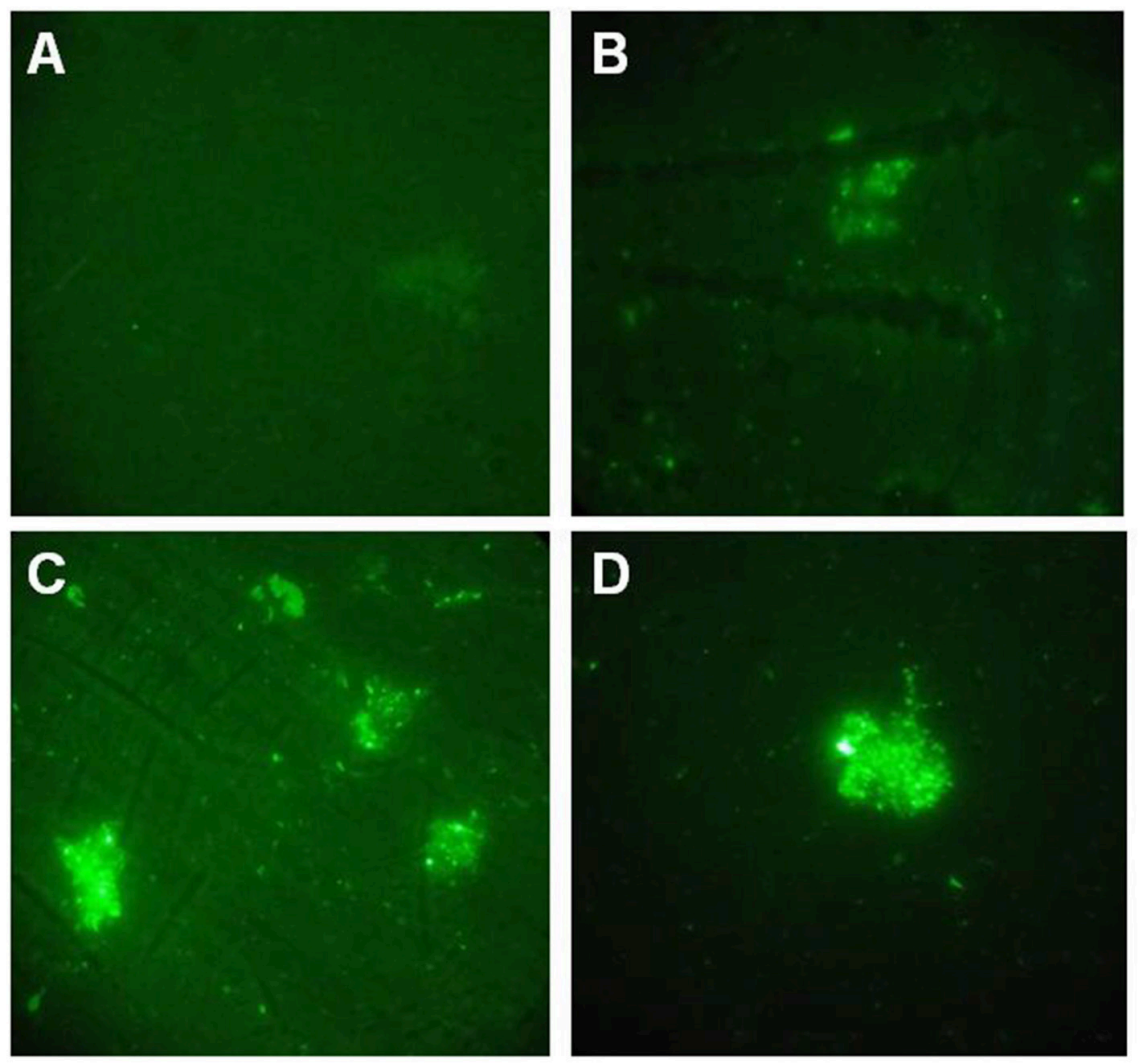
Indirect immunofluorescence assay. **(A)**, PBS immunized serum was used to detect GALT on the cell surface of APP L20 strain; **(B,C)**, GALT immunized serum was used to detect GALT on the cell surface of APP MS71**(B)** and L20 **(C)**; **(D)** Inactivated APP L20 strain immunized serum was used on APP L20 strain as a positive control.

Indirect immunofluorescence method was performed to detect GALT. Fluorescence signal were detected on the cell surface of both of APP MS71 and L20 in the GALT immune serum (Figures 2B,C). At the same time, fluorescence was also detected in the inactivated APP L20 immunized group, which was considered as a positive control (Figure 2D).

### As an *in vivo*-induced antigen, GALT plays an important role of induction of pro-inflammatory cytokines

**Figure 3:**
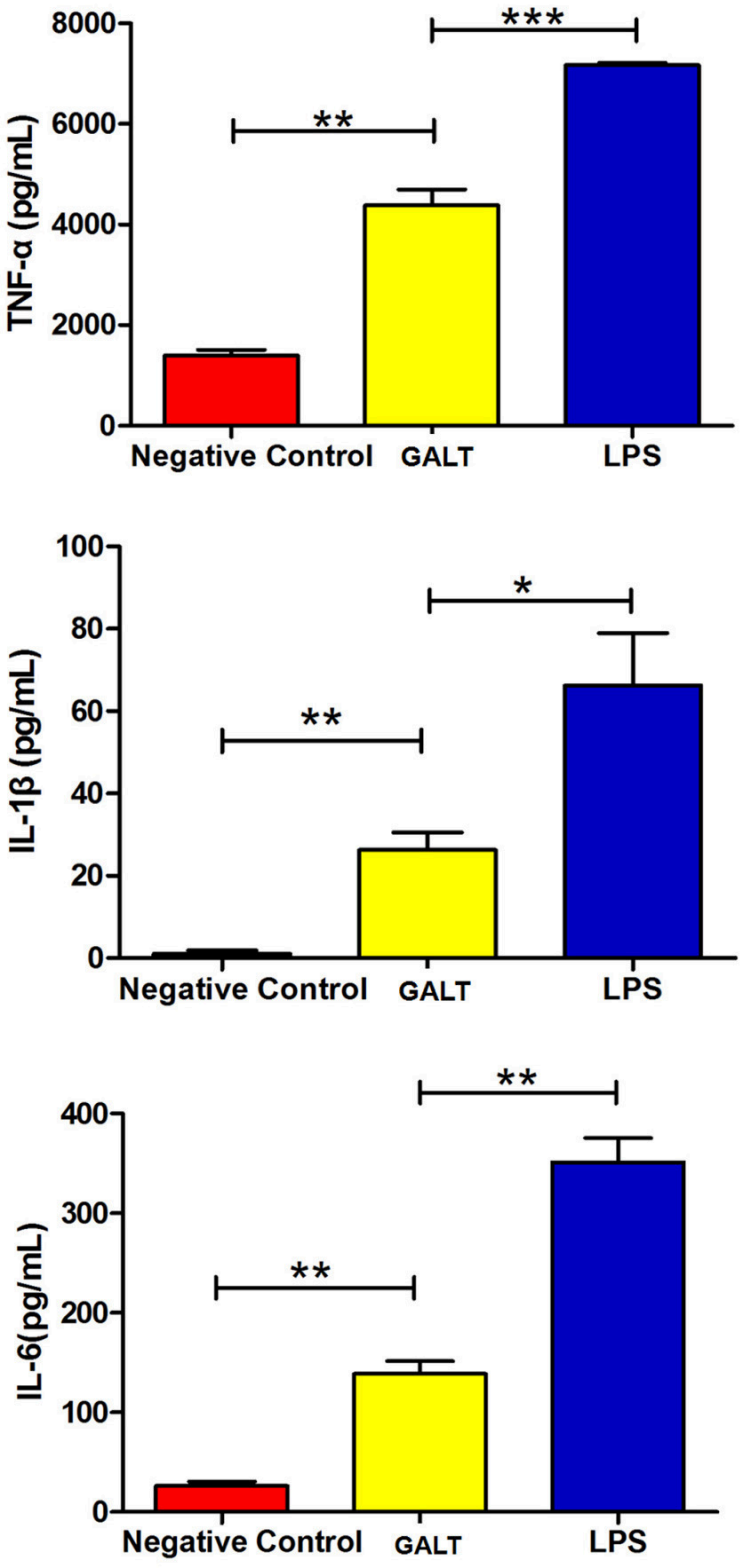
Pro-inflammatory cytokines induced by GALT. Induction of cytokines in Raw 264.7 macrophages by recombinant GALT stimulation. Raw 264.7 macrophages were incubated with 10 μg/mL GALT and 200 ng/mL LPS (positive control) for 12 h, as well as single culture media (negative control). Protein levels of TNF-α, IL-1β, IL-6 in the culture supernatants were determined by ELISA.

To analyze the function of GALT antigen in pathogenesis, pro-inflammatory cytokine responses were measured. After mouse Raw 264.7 macrophages were treated with GALT recombinant protein, secreted cytokines (TNF-α, IL-1β, and IL-6) in the supernatant of cell culture were detected using an ELISA kit. Low secretion levels of TNF-α, IL-1β, and IL-6 were obtained in the negative control (Treated with PBS) (Figure 3). After treatment with GALT, the secretion level of 3 cytokines in the supernatant of cell culture was significantly up-regulated compared with the negative control (*P* < 0.01). LPS (200 ng) was the positive control, which promoted the up-regulation of the production of these cytokines.

### Transcriptional levels of *Tnf-α* and *Il-6* were significantly up-regulated by GALT

**Figure 4:**
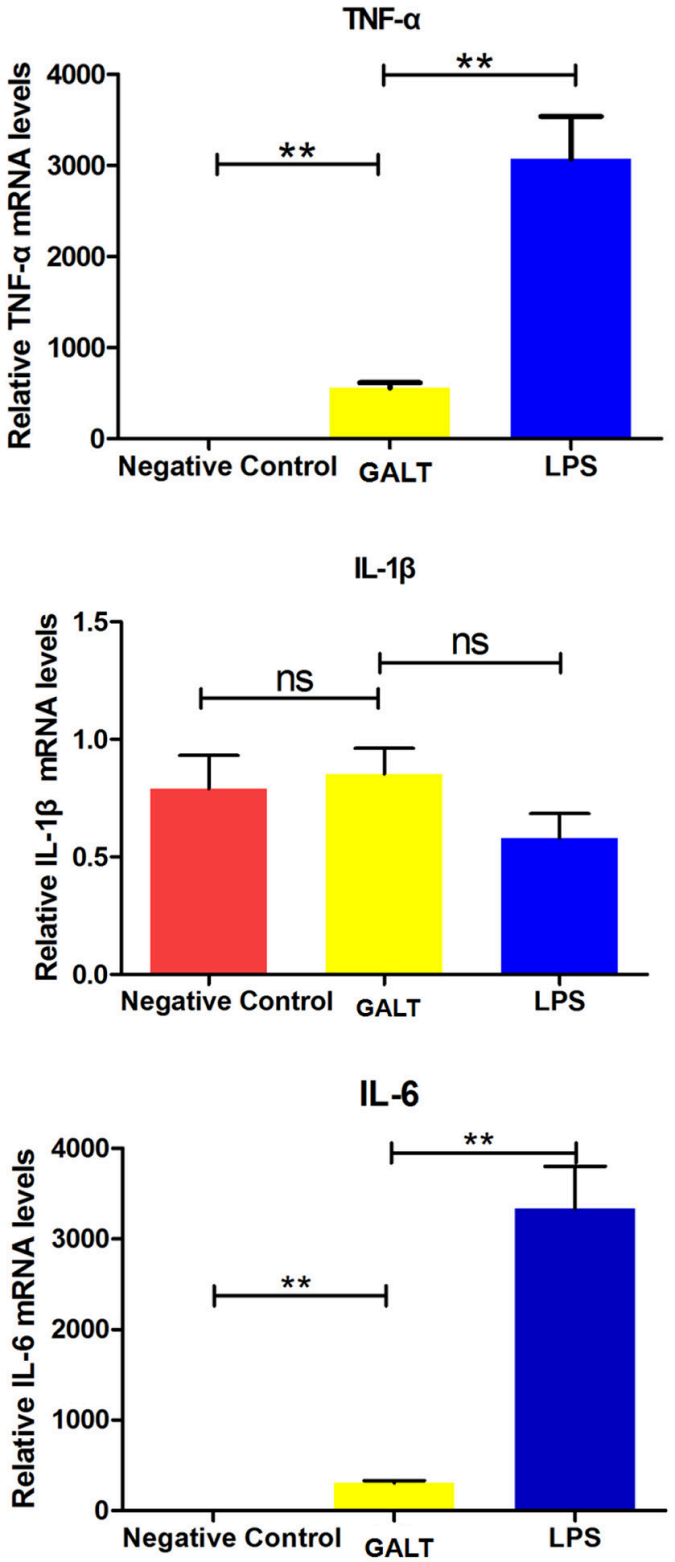
Real-time RT-PCR. Induction of cytokines in Raw 264.7 macrophages by recombinant GALT stimulation. Raw 264.7 macrophages were incubated with 10 μg/mL GALT and 200 ng/mL LPS (positive control) for 12 h, as well as single culture media (negative control). Quantification analysis was conducted to analyze the transcriptional levels of TNF-α, IL-1β, IL-6. β-actin was chosen to be a reference gene in relatively quantification analysis.

In order to uncover the mechanism of GALT on up-regulation function of pro-inflammatory cytokines, transcriptional levels were analyzed by real-time PCR. Beta-actin gene of Raw 246.7 cells was used as a reference gene for quantitative PCR. Both mRNA levels of *Tnf-*α and *Il-6* in Raw 246.7 were significantly up-regulated after treatment with GALT compared with negative control (Figure 4, *P* < 0.01). A low up-regulation of transcription level was observed in *Il-1b* (Figure 4, statistically not significant).

### TLR4 and TLR2 play regulatory roles on expression of pro-inflammatory cytokines in macrophages

**Figure 5:**
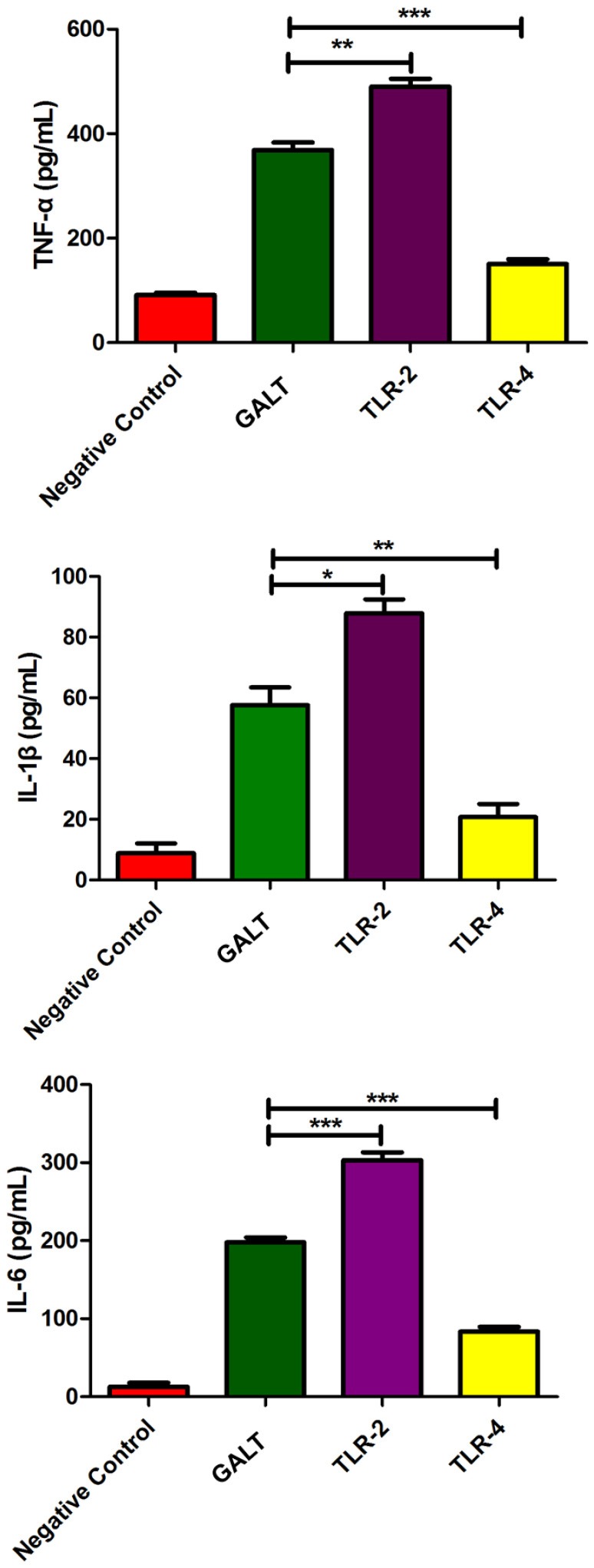
Analysis of the recognition receptor of GALT. Anti-TLR2 and anti-TLR4 antibodies were used to block the corresponding Toll-like receptor. Secretion of pro-inflammatory cytokines were detected to analyze the function of TLR2 and TLR4.

To explore if Toll like receptors mediate the GALT induced up-regulation expression of pro-inflammatory cytokines, anti-TLR4 and anti-TLR2 antibodies were applied in the cell culture to block macrophage cell surface receptors. In the anti-TLR2 group, secreted levels of all of the 3 pro-inflammatory cytokines TNF-α (*P* < 0.01), IL-1β (*P* < 0.05), and IL-6 (*P* < 0.001) were up-regulated significantly (Figure 5). Nevertheless, all of the 3 pro-inflammatory cytokines were all down-regulated significantly after TLR4 of macrophage was blocked with anti-TLR4 compared with the GALT group (Figure 5). In summary, TLR4 and TLR2 mediated GALT induced expression of pro-inflammatory cytokines in macrophages.

### Production of pro-inflammatory cytokines through MAPKs signal pathway

**Figure 6:**
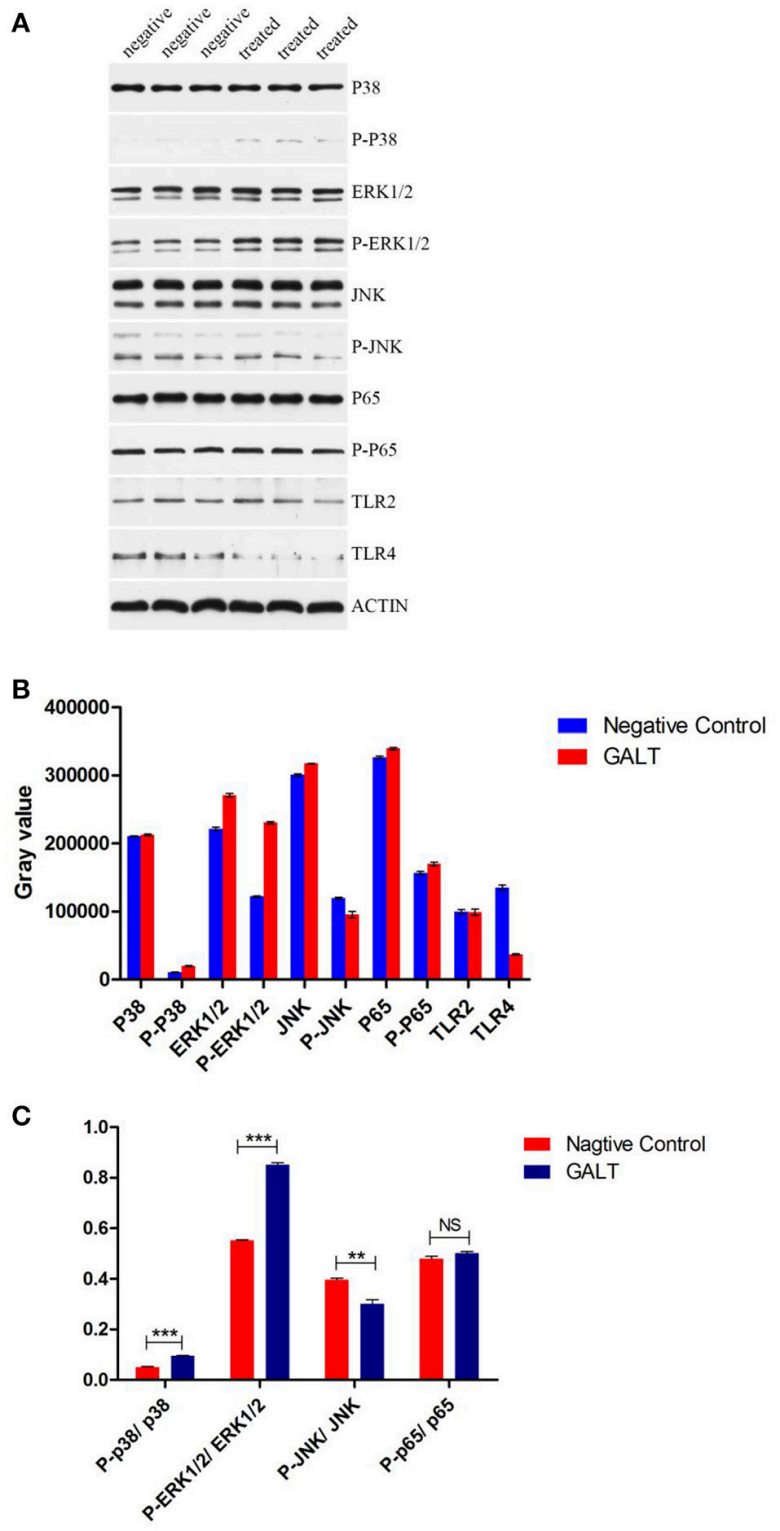
Detection of the signaling molecule involved in cytokine secretion. **(A)** Western Blot Analysis. **(B)** gray intensity. **(C)** Ratios between phosphorylated activated signal molecular and signal molecular. Signaling molecule in MAPKs and NK-κB signaling pathway were detected by western blotting. Different between GALT group and negative control group were also analyzed by gray intensity.

**Figure 7:**
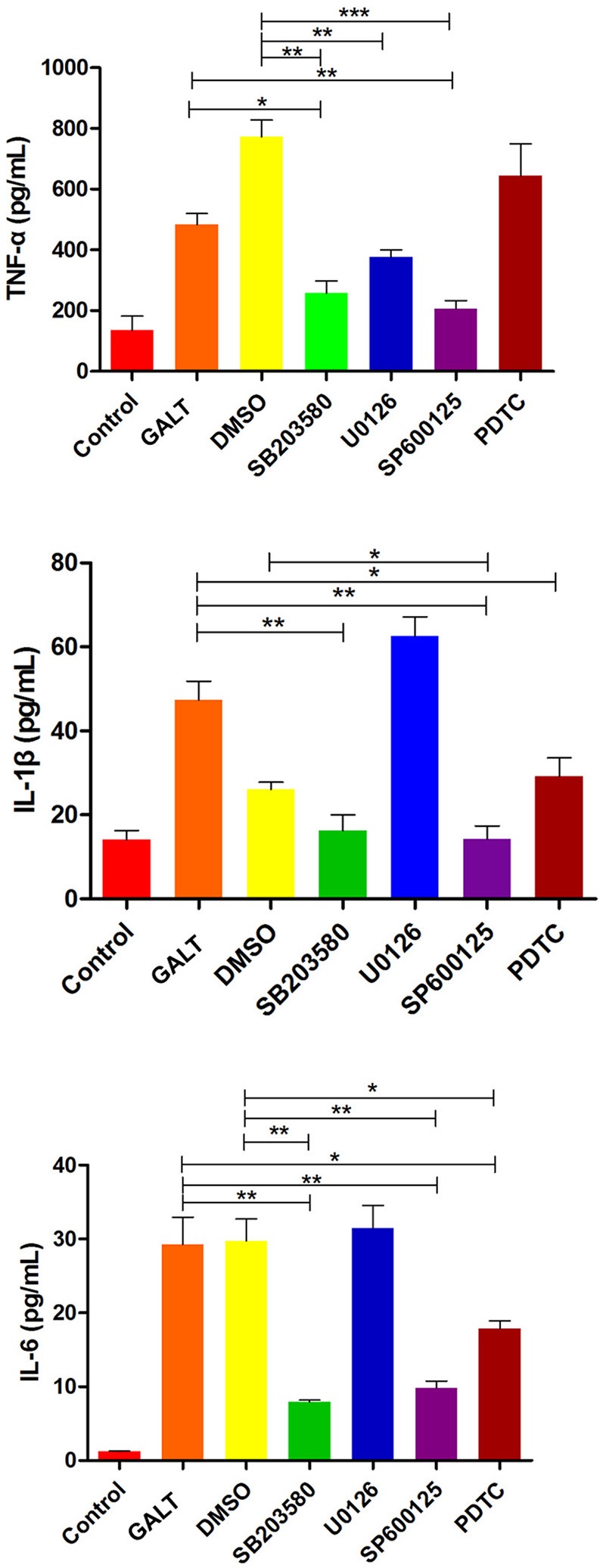
Effects of inhibitor on the pro-inflammatory cytokines secretion induced by GALT. To make clear how GALT protein promoted cytokines secretion, inhibitors SB203580 (p38 inhibitor), U0126 (an ERK1/2 inhibitor), SP600125 (JNK inhibitor), and PDTC (NF-kB inhibitor) were applied to inhibit the corresponding MAPKs and NF-κB cell signaling pathways.

**Figure 8:**
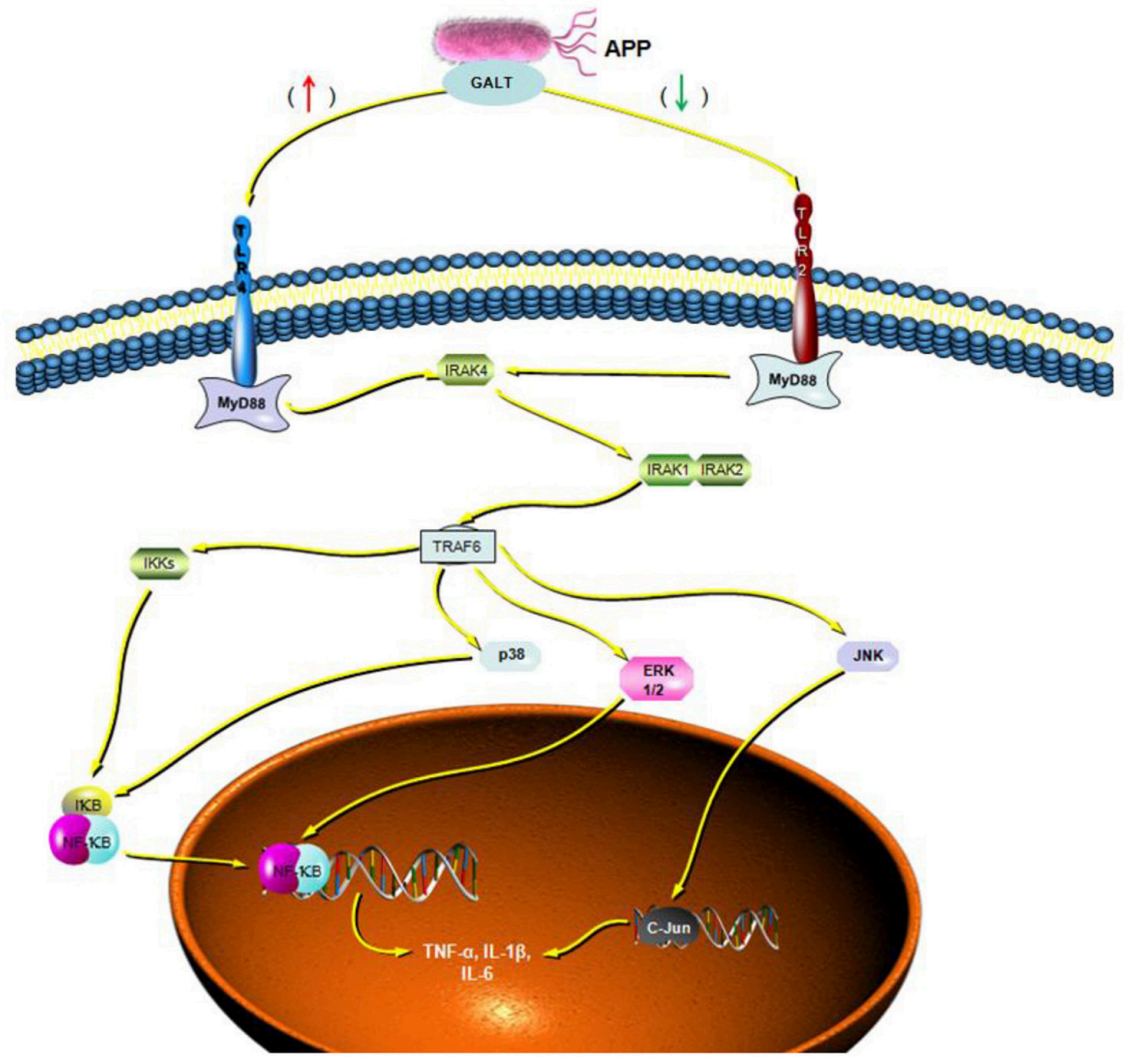
Cellar signal transduction pathway induced by GALT. Based on the results of signaling pathway analyzed induced by GALT, an illustration was made depicting the activities of GALT.

To verify the hypothesis that GALT induce pro-inflammatory cytokines via MAPKs and NF-κB pathway, we performed a western blotting analysis to measure the phosphorylation of ERK 1/2 MAPK and NF-κB in Raw 264.7 cells induced by GALT stimulation (Figure 6). The results showed that the ratios of phosphorylation of ERK 1/2 and p38 MAPK were enhanced, whereas that of JNK was decreased. In the present study, western blot was performed to determine whether GALT stimulates NF-κB signaling. Analysis of macrophage whole cell proteins showed that GALT treatment activated NF-κB(p65). The phosphorylation of NF-κB was increased slightly. Expression of TLR2 and TLR4 in macrophages was tested (Figure 6). After treatment with GALT, expression of TLR2 was down-regulated slightly. However, TLR4 was significantly down-regulated in Raw 264.7 compared with the negative control (Figure 6). β-actin was used as a control in the process of western blot. To make clear how GALT protein promoted cytokines secretion, inhibitors SB203580 (p38 inhibitor), U0126 (an ERK1/2 inhibitor), SP600125 (JNK inhibitor), and PDTC(NF-kB inhibitor) were applied to inhibit the corresponding MAPKs and NF-κB cell signaling pathways. Cells were pretreated with inhibitors and the levels of TNF-α, IL-1β and IL-6 in the supernatants were quantified by ELISA (Figure 7). As shown in Figure 6, the p38 and JNK MAPK inhibitor significantly decreased GALT-induced cytokine production, and the NF-κB inhibitor (PDTC) induced a lower degree of reduction. This result suggested that the GALT-induced cytokine production likely primarily depends on the phosphorylation of ERK1/2 MAPK. The three cytokines tested in this manuscript seem to be produced through different signaling pathways. TNF-α is dependent on p38, ERK1/2 and JNK MAPKs, but not NF-κB. IL-1β and IL-6 seem to be dependent on p38, JNK and NF-κB, but not ERK1/2. Based on the factors affected by GALT, the cell signal transduction pathway was mapped out using PowerPoint 2007 (Microsoft Office, USA) software (Figure 8).

### GALT protein sequences are conserved in different pathogens in family *Pasteurella*

**Figure 9:**
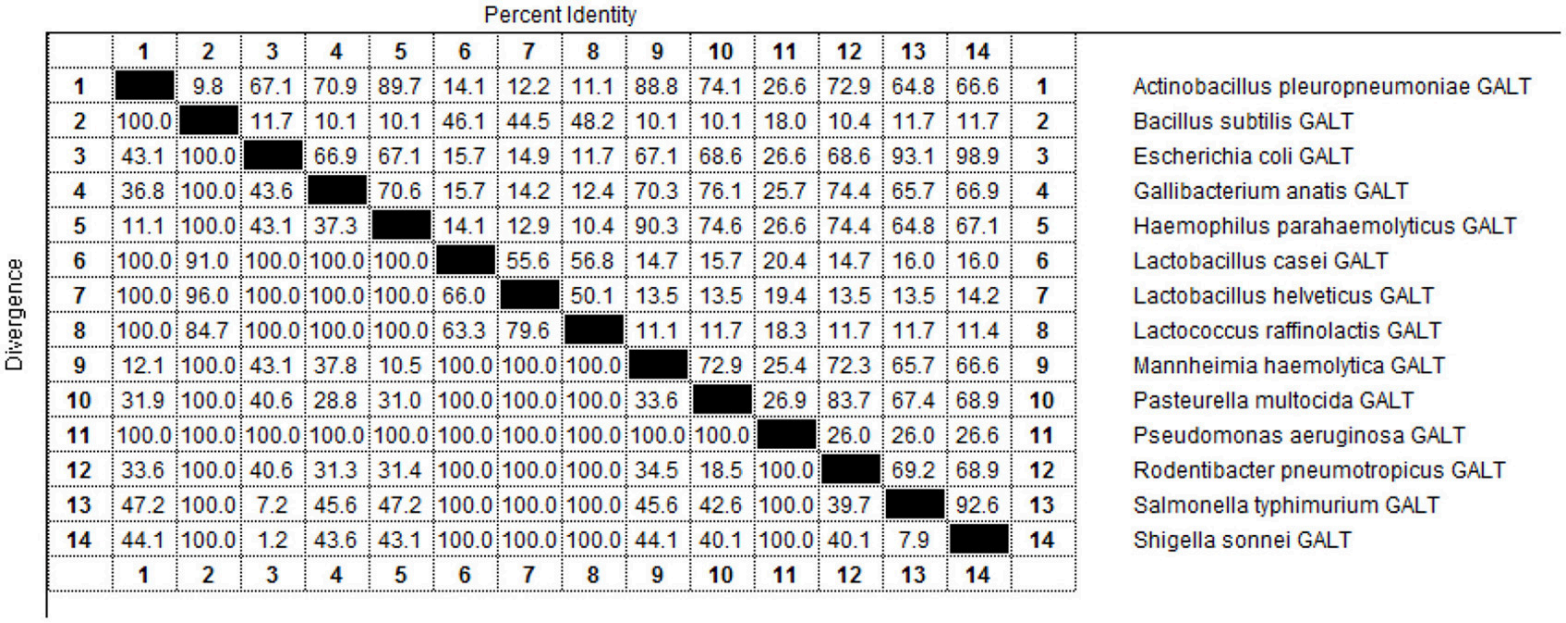
Percent identity of the amino acid sequences of GALT in different pathogens.

As indicated by the results of BLAST-P, GALT is widely distributed among different microbes and amino acid sequences are conserved (Figure 9). Fourteen pathogens in were selected for sequencing and structural analysis. According to the results of MegAlign, there were high identities among the amino acid sequences of *Actinobacillus pleuropneumoniae, Escherichia coli, Gallibacterium anatis, Haemophilus parahaemolyticus, Mannheimia haemolytica, Pasteurella multocida, Rodentibacter pneumotropicus, Salmonella typhimurium*, and *Shigella sonnei* ranging from 64.8 to 98.9 % (Figure 9). A PredictProtein online open server was applied to analyze the second structure of GALT protein from fourteen different pathogens (Figure S1) (Yachdav et al., 2014). Protein interaction sites in GALT protein from different pathogens except for *Bacillus subtilis, Lactobacillus casei, Lactobacillus helveticus, Lactococcus raffinolactis* were found to be similar (Figure S1, red square). In the nine bacteria with high identities, GALT binding sites were distributed in all the sequences, but appeared most frequently in the AA38 to AA114 frame. Five main helices were identified in 9 sequences at the same location (crimson band). The analysis results using Solvent Accessibility program demonstrated that these 9 sequences possessed highly similar surface exposed and membrane buried domains (Figure S1). All of these 9 amino acid sequences contain 1 transmembrane helix located within AA220 to AA240 (purple band). Three main similar disordered regions located in the two ends of the sequences in 9 proteins (green band).

## Discussion

APP is a pathogen of swine, causing acute bacterial infection. In the pathogenesis of many bacterial infectious diseases, adhesion and invasion of pathogenic bacteria are the crucial processes in the establishment of infection. In this study, we found that GALT-induces secretion of pro-inflammatory cytokines TNF-α, IL-1β, and IL-6 through the activation of p38, ERK1/2, and JNK MAPKs signal transduction pathway in macrophages.

Bacterial death induces endotoxin release into circulation, often resulting in endotoxemia. In this stage of bacterial infection, pro-inflammatory cytokines such as TNF-α, IL-1β, and IL-6 are elevated (Choi et al., 1999; Cho and Chae, 2002), playing a crucial role in the pathogenesis of acute bacterial infection (Li et al., 2018). In this study, secretion of pro-inflammatory cytokines TNF-α, IL-1β, and IL-6 from macrophages were significantly (P < 0.01) promoted after stimulation with GALT (Figure 3). GALT was identified as an *in vivo*-induced antigen, which can be up-regulated several thousand fold *in vivo* compared with *in vitro* (Zhang et al., 2015a). The *gal*T has been shown be crucial gene for bacterial pathogen colonization and is associated with the virulence of the pathogens (Marianne et al., 1994; Ho and Waldor, 2007).

Toll-like receptors play a crucial role in the process of interactions of hosts and microbes (Sasai and Yamamoto, 2013). Lipoproteins are crucial pathogenic components for all bacterial pathogens (BLPs), and trigger natural immunity. BLPs can induce apoptosis in THP-1 monocytic cells through human Toll-like receptor-2 interaction (hTLR2) (Aliprantis et al., 1999). Moreover, bacterial components such as LPS, LTA, PG, porins, and lipoarabinomannan all were recognized by TLR4 or TLR2 (Akira et al., 2006; Selvaraj et al., 2015; Park et al., 2017). Newly discovered components of bacterial pathogens have also demonstrated interaction with TLR2 via an antibody blocking assay; these may serve as ligands of TLR2 (Zhang et al., 2015b). In present study, TLR2 and TLR4 were demonstrated to be the receptor of the antigen GALT of APP and the changes in the levels of cytokines were significantly altered post TLR2 or TLR4 Ig binding. First, the expression of cytokines was decreased significantly after TLR4 binding was blocked. Nevertheless, the level of expressed cytokines were increased significantly after TLR2 binding was blocked. It can be speculated that both of TLR2 and TLR4 combine with the antigen GALT used in this study. The combination of GALT with TLR2 may involve negative control of cytokine secretion. After TLR4 was blocked on macrophages, the concentrations of cytokines in the cell cultural supernatant were found to decrease. In the present study, TLR-2 and TLR-4 are involved in positive and negative control of cytokine production, respectively.

Following pathogen infection, pattern recognition receptors on the cell surface or in the cytoplasm of innate immune cells are stimulated and MAPK signal pathways are activated, including ERK, p38, and JNK subfamilies (Arthur and Ley, 2013). In our present study, signal transduction pathway p38 and JNK MAPKs were demonstrated to play primary roles in the pro-inflammatory response induced by GALT stimulation in macrophages (Figure 6). Combined with activation of nuclear factor-κB, MAPKs signal pathways promote the expression of multiple genes to regulate the inflammatory response (Iwasaki and Medzhitov, 2010; Arthur and Ley, 2013). Inhibitor assays in present study demonstrated that pro-inflammatory responses induced by GALT includes stimulation of nuclear factor-kB, which is a transcriptional activator of multiple host defense genes (Figures 7, 8).

The *gal*T, a member of the *gal* gene cluster, an important group associated with virulence, survival in host and so on, is widely distributed in different pathogens(Lai et al., 2001; Nesper et al., 2001; Oechslin et al., 2017; Pires et al., 2017). GALT also distributed in a variety of other gram-negative and gram-positive bacteria such as *Escherichia coli, Pseudomonas aeruginosa, Shigella sonnei*, and *Lactobacillus helveticus* (Ketner and Campbell, 1974; Schümperli et al., 1982; Torino et al., 2005; Ho and Waldor, 2007; Caboni et al., 2015; Oechslin et al., 2017). From the results of BLAST, amino acid sequences of the pathogens in family *Pasteurella* showed a high level of identity. As shown in the results of Predict Protein online open server analysis, all the sequences possess a similar amino acid secondary structures (Figure S1).

We demonstrated GALT protein of APP was a vital antigen, which could promote the secretion of pro-inflammatory cytokines. This process includes cell signaling pathways like P38, ERK1/2, JNK MAPKs, and NF-κB. Both TLR2 and TLR4 are receptors of GALT antigens, whereas they played negative and positive roles (respectively) in the process of induction of pro-inflammatory cytokines. GALT is a conserved antigen among various pathogens, and warrants further study in efforts aimed at elucidating details concerning pathogenesis and pathogen interaction with the host cells.

## Author contributions

FZ, QZ, and SC conceived and designed the experiments. FZ, QZ, JT, XW, CM, YW, QY, and RW performed the experiments. FZ, Y-FC, XiaH, YH, XinH, CM, and XM analyzed the data. FZ, SC, and Y-FC wrote the paper.

### Conflict of interest statement

The authors declare that the research was conducted in the absence of any commercial or financial relationships that could be construed as a potential conflict of interest. The reviewer VT and handling Editor declared their shared affiliation.

## References

[B1] Akira S. Takeda K. Kaisho T. (2001). Toll-like receptors: critical proteins linking innate and acquired immunity. Nat. Immunol. 2, 675–680. 10.1038/90609 11477402

[B2] Akira S. Uematsu S. Takeuchi O. (2006). Pathogen recognition and innate immunity. Cell 124, 783–801. 10.1016/j.cell.2006.02.015 16497588

[B3] Aliprantis A. O. Yang R.-B. Mark M. R. Suggett S. Devaux B. Radolf J. D.. (1999). Cell activation and apoptosis by bacterial lipoproteins through toll-like receptor-2. Science 285, 736–739. 10.1126/science.285.5428.736 10426996

[B4] Arthur J. S. Ley S. C. (2013). Mitogen-activated protein kinases in innate immunity. Nat. Rev. Immunol. 13, 679–692. 10.1038/nri3495 23954936

[B5] Caboni M. Pedron T. Rossi O. Goulding D. Pickard D. Citiulo F.. (2015). An O antigen capsule modulates bacterial pathogenesis in Shigella sonnei. PLoS Pathog. 11:e1004749. 10.1371/journal.ppat.1004749 25794007 PMC4368438

[B6] Chai Y. Beauregard P. B. Vlamakis H. Losick R. Kolter R. (2012). Galactose metabolism plays a crucial role in biofilm formation by Bacillus subtilis. MBio 3, e00184–e00112. 10.1128/mBio.00184-12 22893383 PMC3419520

[B7] Chen Z. W. Chien M. S. Chang N. Y. Chen T. H. Wu C. M. Huang C.. (2011). Mechanisms underlying Actinobacillus pleuropneumoniae exotoxin ApxI induced expression of IL-1beta, IL-8 and TNF-alpha in porcine alveolar macrophages. Vet. Res. 42:25. 10.1186/1297-9716-42-25 21314908 PMC3041667

[B8] Cho W. S. Chae C. (2002). Expression of nitric oxide synthase 2 and tumor necrosis factor in swine naturally infected with *Actinobacillus pleuropneumoniae*. Vet. Pathol. 39, 27–32. 10.1354/vp.39-1-27 12102216

[B9] Choi C. Kwon D. Min K. Chae C. (1999). *In-situ* hybridization for the detection of inflammatory cytokines (IL-1, TNF-a and IL-6) in pigs naturally infected with *Actinobacillus pleuropneumoniae*. J. Comp. Pathol. 121, 349–356. 10.1053/jcpa.1999.0332 10542124

[B10] Fuller T. E. Martin S. Teel J. F. Alaniz G. R. Kennedy M. J. Lowery D. E. (2000). Identification of *Actinobacillus pleuropneumoniae* virulence genes using signature-tagged mutagenesis in a swine infection model. Microb. Pathog. 29, 39–51. 10.1006/mpat.2000.0364 10873489

[B11] Handfield M. Brady L. J. Progulske-Fox A. Hillman J. D. (2000). IVIAT: a novel method to identify microbial genes expressed specifically during human infections. Trends Microbiol. 8, 336–339. 10.1016/S0966-842X(00)01775-3 10878769

[B12] Ho T. D. Waldor M. K. (2007). Enterohemorrhagic *Escherichia coli* O157:H7 gal mutants are sensitive to bacteriophage P1 and defective in intestinal colonization. Infect. Immun. 75, 1661–1666. 10.1128/IAI.01342-06 17158899 PMC1865682

[B13] Iwasaki A. Medzhitov R. (2010). Regulation of adaptive immunity by the innate immune system. Science 327, 291–295. 10.1126/science.1183021 20075244 PMC3645875

[B14] Kats A. Norgard M. Wondimu Z. Koro C. Concha Quezada H. Andersson G.. (2016). Aminothiazoles inhibit RANKL- and LPS-mediated osteoclastogenesis and PGE2 production in RAW 264.7 cells. J. Cell Mol. Med. 20, 1128–1138. 10.1111/jcmm.12814 26987561 PMC4882984

[B15] Kawai T. Akira S. (2010). The role of pattern-recognition receptors in innate immunity: update on Toll-like receptors. Nat. Immunol. 11, 373–384. 10.1038/ni.1863 20404851

[B16] Ketner G. Campbell A. (1974). A deletion mutation placing the galactokinase gene of *Escherichia coli* under control of the biotin promoter. Proc. Natl. Acad. Sci. U.S.A. 71, 2698–2702. 10.1073/pnas.71.7.2698 4368323 PMC388535

[B17] Kurt-Jones E. A. Popova L. Kwinn L. Haynes L. M. Jones L. P. Tripp R. A.. (2000). Pattern recognition receptors TLR4 and CD14 mediate response to respiratory syncytial virus. Nat. Immunol. 1, 398–401. 10.1038/80833 11062499

[B18] Lai Y. C. Peng H. L. Chang H. Y. (2001). Identification of genes induced *in vivo* during *Klebsiella pneumoniae* CG43 infection. Infect. Immun. 69, 7140–7145. 10.1128/IAI.69.11.7140-7145.2001 11598090 PMC100105

[B19] Li B. Fang J. Zuo Z. Yin S. He T. Yang M.. (2018). Activation of porcine alveolar macrophages by *Actinobacillus pleuropneumoniae* lipopolysaccharide via the toll-like receptor 4/NF-B-mediated pathway. Infect. Immun. 86, e00642–e00617. 10.1128/IAI.00642-17 29229731 PMC5820947

[B20] Marianne M. Peter B. Peter B. Klaus G. (1994). Genetics of galactose metabolism of *Erwinia amylovora* and its influence on polysaccharide synthesis and virulence of the fire blight pathogen. J. Bacteriol. 176, 450–459. 10.1128/jb.176.2.450-459.1994 7507102 PMC205069

[B21] Medzhitov R. Horng T. (2009). Transcriptional control of the inflammatory response. Nat. Rev. Immunol. 9, 692–703. 10.1038/nri2634 19859064

[B22] Medzhitov R. Preston-Hurlburt P. Janeway C. A. Jr. (1997). A human homologue of the Drosophila Toll protein signals activation of adaptive immunity. Nature 388, 394–397. 10.1038/41131 9237759

[B23] Meyer C. Hoffmann C. Haas R. Schubert S. (2015). The role of the galU gene of uropathogenic *Escherichia coli* in modulating macrophage TNF-alpha response. Int. J. Med. Microbiol. 305, 893–901. 10.1016/j.ijmm.2015.09.004 26481693

[B24] Nesper J. Lauriano C. M. Klose K. E. Kapfhammer D. Kraiss A. Reidl J. (2001). Characterization of *Vibrio cholerae* O1 El tor galU and galE mutants: influence on lipopolysaccharide structure, colonization, and biofilm formation. Infect. Immun. 69, 435–445. 10.1128/IAI.69.1.435-445.2001 11119535 PMC97901

[B25] Oechslin F. Piccardi P. Mancini S. Gabard J. Moreillon P. Entenza J. M.. (2017). Synergistic interaction between phage therapy and antibiotics clears *Pseudomonas aeruginosa* infection in endocarditis and reduces virulence. J. Infect. Dis. 215, 703–712. 10.1093/infdis/jiw632 28007922 PMC5388299

[B26] Park S. Shin H. J. Shah M. Cho H. Y. Anwar M. A. Achek A.. (2017). TLR4/MD2 specific peptides stalled *in vivo* LPS-induced immune exacerbation. Biomaterials 126, 49–60. 10.1016/j.biomaterials.2017.02.023 28254693

[B27] Peddireddy V. Doddam S. N. Qureshi I. A. Yerra P. Ahmed N. (2016). A putative nitroreductase from the DosR regulon of *Mycobacterium tuberculosis* induces pro-inflammatory cytokine expression via TLR2 signaling pathway. Sci. Rep. 6:24535. 10.1038/srep24535 27094446 PMC4837367

[B28] Pires D. P. Dotsch A. Anderson E. M. Hao Y. Khursigara C. M. Lam J. S.. (2017). A genotypic analysis of five, *P. aeruginosa* strains after biofilm infection by phages targeting different cell surface receptors. Front. Microbiol. 8:1229. 10.3389/fmicb.2017.01229 28713356 PMC5492357

[B29] Priebe G. P. Dean C. R. Zaidi T. Meluleni G. J. Coleman F. T. Coutinho Y. S.. (2004). The galU Gene of *Pseudomonas aeruginosa* is required for corneal infection and efficient systemic spread following pneumonia but not for infection confined to the lung. Infect. Immun. 72, 4224–4232. 10.1128/IAI.72.7.4224-4232.2004 15213167 PMC427465

[B30] Ramjeet M. Cox A. D. Hancock M. A. Mourez M. Labrie J. Gottschalk M.. (2008). Mutation in the LPS outer core biosynthesis gene, galU, affects LPS interaction with the RTX toxins ApxI and ApxII and cytolytic activity of *Actinobacillus pleuropneumoniae* serotype 1. Mol. Microbiol. 70, 221–235. 10.1111/j.1365-2958.2008.06409.x 18713318

[B31] Sasai M. Yamamoto M. (2013). Pathogen recognition receptors: ligands and signaling pathways by Toll-like receptors. Int. Rev. Immunol. 32, 116–133. 10.3109/08830185.2013.774391 23570313

[B32] Schümperli D. Mckenney K. Sobieski D. A. Rosenberg M. (1982). Translational coupling at an lntercistronic boundary of the *Escherichia coli* galactose operon. Cell 30, 865–871. 10.1016/0092-8674(82)90291-4 6754091

[B33] Selvaraj V. Nepal N. Rogers S. Manne N. D. Arvapalli R. Rice K. M.. (2015). Inhibition of MAP kinase/NF-kB mediated signaling and attenuation of lipopolysaccharide induced severe sepsis by cerium oxide nanoparticles. Biomaterials 59, 160–171. 10.1016/j.biomaterials.2015.04.025 25968464 PMC4565726

[B34] Takeda K. Akira S. (2005). Toll-like receptors in innate immunity. Int. Immunol. 17, 1–14. 10.1093/intimm/dxh186 15585605

[B35] Tang S. Chen T. Yu Z. Zhu X. Yang M. Xie B.. (2014). RasGRP3 limits Toll-like receptor-triggered inflammatory response in macrophages by activating Rap1 small GTPase. Nat. Commun. 5:4657. 10.1038/ncomms5657 25118589 PMC4143924

[B36] Torino M. I. Mozzi F. Font De Valdez G. (2005). Exopolysaccharide biosynthesis by *Lactobacillus helveticus* ATCC 15807. Appl. Microbiol. Biotechnol. 68, 259–265. 10.1007/s00253-004-1865-2 15660218

[B37] Vigil P. D. Alteri C. J. Mobley H. L. (2011). Identification of *in vivo*-induced antigens including an RTX family exoprotein required for uropathogenic *Escherichia coli* virulence. Infect. Immun. 79, 2335–2344. 10.1128/IAI.00110-11 21422188 PMC3125824

[B38] Wong S. M. Akerley B. J. (2005). Environmental and genetic regulation of the phosphorylcholine epitope of *Haemophilus influenzae* lipooligosaccharide. Mol. Microbiol. 55, 724–738. 10.1111/j.1365-2958.2004.04439.x 15660999

[B39] Wu C. M. Chen Z. W. Chen T. H. Liao J. W. Lin C. C. Chien M. S.. (2011). Mitogen-activated protein kinases p38 and JNK mediate *Actinobacillus pleuropneumoniae* exotoxin ApxI-induced apoptosis in porcine alveolar macrophages. Vet. Microbiol. 151, 372–378. 10.1016/j.vetmic.2011.03.033 21550186

[B40] Yachdav G. Kloppmann E. Kajan L. Hecht M. Goldberg T. Hamp T.. (2014). PredictProtein—an open resource for online prediction of protein structural and functional features. Nucleic Acids Res. 42, W337–W343. 10.1093/nar/gku366 24799431 PMC4086098

[B41] Yang F. Ma Q. Lei L. Huang J. Ji Q. Zhai R.. (2014). Specific humoral immune response induced by propionibacterium acnes can prevent *Actinobacillus pleuropneumoniae* infection in mice. Clin. Vaccin.Immunol. 21, 407–416. 10.1128/CVI.00667-13 24429068 PMC3957672

[B42] Zhang F. Cao S. Zhu Z. Yang Y. Wen X. Chang Y. F.. (2016). Immunoprotective efficacy of six *in vivo*-induced antigens against actinobacillus pleuropneumoniae as potential vaccine candidates in murine model. Front. Microbiol. 7:1623. 10.3389/fmicb.2016.01623 27818646 PMC5073529

[B43] Zhang F. Zhang Y. Wen X. Huang X. Wen Y. Wu R.. (2015a). Identification of *Actinobacillus pleuropneumoniae* genes preferentially expressed during infection using *in vivo*-induced antigen technology (IVIAT). J. Microbiol. Biotechnol. 25, 1606–1613. 10.4014/jmb.1504.04007 26059519

[B44] Zhang Q. Yang Y. Yan S. Liu J. Xu Z. Yu J.. (2015b). A novel pro-inflammatory protein of Streptococcus suis 2 induces the Toll-like receptor 2-dependent expression of pro-inflammatory cytokines in RAW 264.7 macrophages via activation of ERK1/2 pathway. Front. Microbiol. 6:178. 10.3389/fmicb.2015.00178 25806027 PMC4353370

