# Supplementary material for: Effective Pro-Inflammatory Induced Activity of GALT, a Conserved Antigen in A. Pleuropneumoniae, Improves the Cytokines Secretion of Macrophage via p38, ERK1/2 and JNK MAPKs Signal Pathway
Source: Front Cell Infect Microbiol. 2018 Sep 25;8:337. doi: 10.3389/fcimb.2018.00337 (PMC6167544; doi:10.3389/fcimb.2018.00337)
Supplement: Supplementary file 1 [file Table_1.DOC]

1. Actinobacillus pleuropneumoniae GALT:

<https://open.predictprotein.org/visual_results?req_id=$1$/vPn5Uzy$nu.r9lAGVMrYQsPTOicMr0>


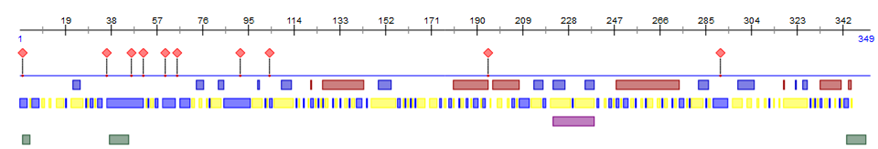


2. Bacillus subtilis GALT:

<https://open.predictprotein.org/visual_results?req_id=$1$qYkjsnqA$wBOW3455pBBaHxxZb1SND>.


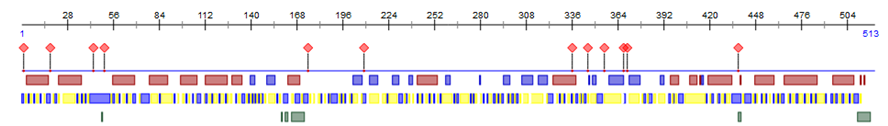


3. Escherichia coli GALT:

<https://open.predictprotein.org/visual_results?req_id=$1$U..DQlR3$reKMEXErVLyl.Uphkwahe1>


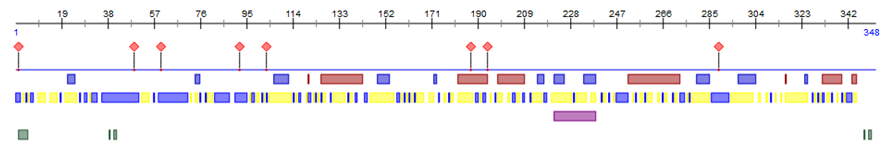


4. Gallibacterium anatis GALT:

<https://open.predictprotein.org/visual_results?req_id=$1$ZYBkK1Ve$fB4EpqZRE2PSZuCiG8rI80>


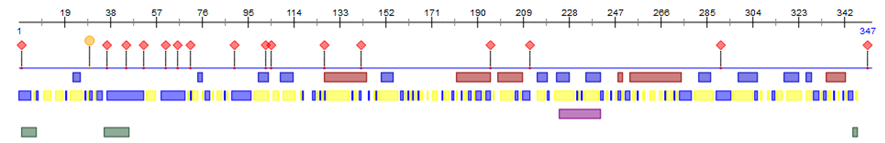


5. Haemophilus parahaemolyticus GALT:

<https://open.predictprotein.org/visual_results?req_id=$1$/5iDAeCO$Sk1k76dayBDEUFv7J40vn>.


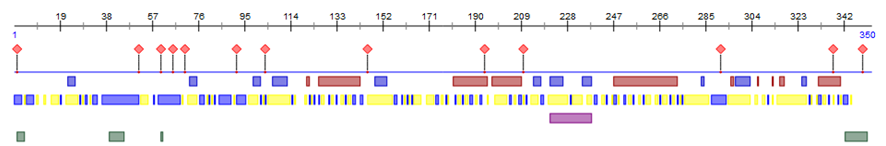


6. Lactobacillus casei GALT:

<https://open.predictprotein.org/visual_results?req_id=$1$JUMiHG1f$rdP0zT5c68twYIY5moLey>.


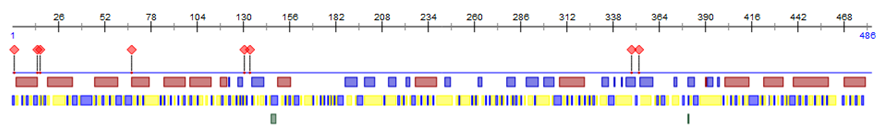


7. Lactobacillus helveticus GALT:

<https://open.predictprotein.org/visual_results?req_id=$1$OvHEBWXl$RMWt50fUgm1sQcrKNM8ZD>.


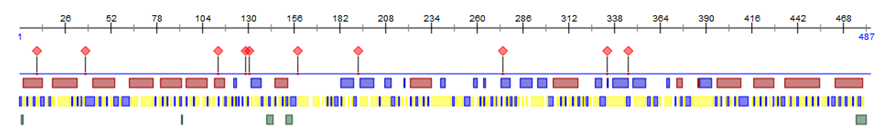


8. Lactococcus raffinolactis GALT:

<https://open.predictprotein.org/get_results?req_id=$1$6u0rFrqi$ctMqoXcX8t9qHIE..BRK8/>


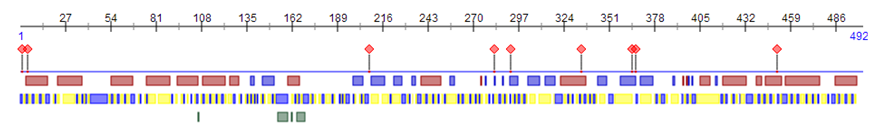


9. Mannheimia haemolytica GALT

<https://open.predictprotein.org/visual_results?req_id=$1$HKz9bmrE$tgnFYrbbRBpTJSUHl8lHr/>


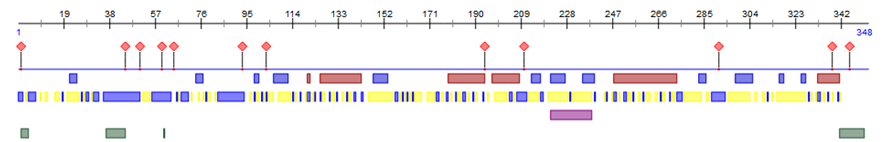


10. Pasteurella multocida GALT:

<https://open.predictprotein.org/visual_results?req_id=$1$8f5GhvnA$SBNI5ZOqMkbUnjOxCdKoY/>


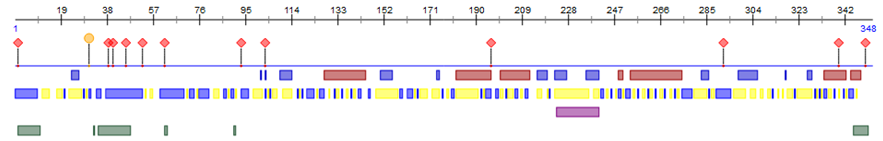


11. Pseudomonas aeruginosa GALT:

<https://open.predictprotein.org/visual_results?req_id=$1$dh2eTKE/$odUaHvjRndScSO9nyiaQk0>


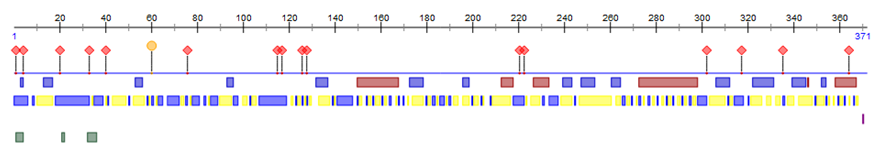


12. Rodentibacter pneumotropicus GALT:

<https://open.predictprotein.org/visual_results?req_id=$1$RLza7RoG$sFfMujE8cJiXsH2UnUYrK/>


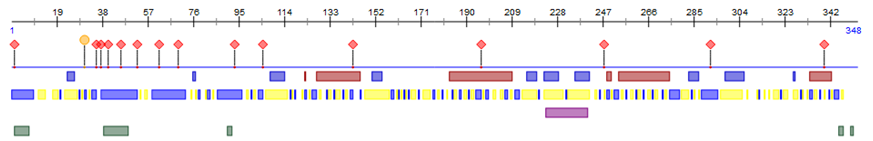


13. Salmonella typhimurium GALT:

<https://open.predictprotein.org/visual_results?req_id=$1$8VSTTgP2$1h13Fz4moB.L.gQ/EX5B91>


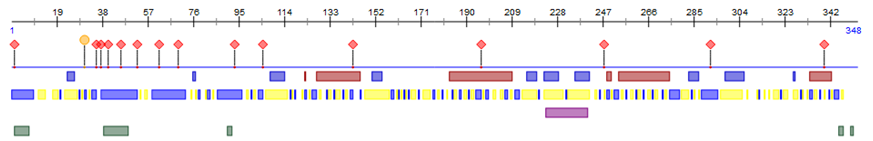


14. Shigella sonnei GALT:

<https://open.predictprotein.org/get_results?req_id=$1$N5IHLvbl$37wPkgO4m.WJUJbD7DzS70>


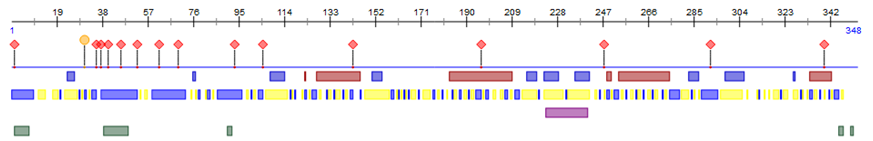


Figure. S1 Results of protein secondary structure prediction of 14 different pathogens. Note: red squares stand for protein binding region(Ofran and Rost, 2007), blue bands in the first line indicate strand (Rost, 2003), crimson bands indicate the presence of helix(Rost, 2003), blue bands in the second line stand for domains exposed to Solvent Accessibility(Bigelow et al., 2004), yellow bands indicate membrane buried domains for Solvent Accessibility(Bigelow et al., 2004), purple bands stand for transmembrane helices(ROST et al., 1996), green bands stand for disordered region(Schlessinger et al., 2009) .

Altschul, S.F., Madden, T.L., Schäffer, A.A., Zhang, J., Zhang, Z., Miller, W., and Lipman, D.J. (1997). Gapped BLAST and PSI-BLAST: a new generation of protein database search programs. *Nucleic Acids Research* 25**,** 3389–3402.

Bigelow, H.R., Petrey, D.S., Liu, J., Przybylski, D., and Rost, B. (2004). Predicting transmembrane beta-barrels in proteomes. *Nucleic Acids Res* 32**,** 2566-2577.

Ofran, Y., and Rost, B. (2007). ISIS: interaction sites identified from sequence. *Bioinformatics* 23**,** e13-16.

Rost, B. (2003). The PredictProtein server. *Nucleic Acids Research* 31**,** 3300-3304.

Rost, B., Fariselli, P., and Casadio, R. (1996). Topology prediction for helical transmembrane proteins at 86% accuracy. *Protein Science* 5**,** 1704-1718.

Schlessinger, A., Punta, M., Yachdav, G., Kajan, L., and Rost, B. (2009). Improved disorder prediction by combination of orthogonal approaches. *PLoS One* 4**,** e4433.
